# Evaluation of management of desmoids tumours associated with familial adenomatous polyposis in Dutch patients

**DOI:** 10.1038/bjc.2011.50

**Published:** 2011-03-01

**Authors:** S Bhandari, A Sinha, S K Clark

**Affiliations:** 1 Polyposis Registry, St Mark's Hospital, Watford Road, Harrow, Middlesex HA1 3UJ, UK

**Sir,**

We read with interest the article by Nieuwenhuis *et al*, ‘Evaluation of management of desmoids tumours associated with familial adenomatous polyposis in Dutch patients’ (Nieuwenhuis *et al*, 2011). Similar studies have been performed in other polyposis registries (Church, 1995; Clark and Phillips, 1996). Because of the rarity of these tumours and their variable course (30% growth and regression, 10% aggressive growth, 50% stable course, 10% spontaneous resolution) (Church, 1995), it is difficult to assess the effect of any one particular therapy.

Although the study by Nieuwenhuis *et al* provides valuable information on this rare condition in one cohort of patients, a number of issues are raised. It is not clear from the presented data what the indications were for ‘primary surgery’ for intra-abdominal tumours. In the introduction, the authors describe a ‘step-wise approach’ to managing desmoids, starting with NSAIDs and antioestrogens, and escalating treatment if disease progresses (Sturt and Clark, 2006). It is not clear whether those undergoing surgery had medical therapy previously or not, and, if not, why they had not been managed using this accepted approach. Generally surgery is reserved for more problematic intra-abdominal desmoids, those that do not respond to medical therapy. It is unclear from this paper exactly what operations were performed. Sometimes it is only possible to bypass or proximally de-function the bowel obstructed by the tumour or to drain desmoid necrosis. Although it is useful to see the outcomes following surgical or medical therapy, a comparison such as the one presented here is only valid if the same indications are used for both treatments, which is unlikely to be the case here.

Progress-free period has been defined as the period from identification of desmoids to the development of symptoms. Certainly, in intra-abdominal desmoids it would be difficult to attribute all symptoms to desmoid disease. How the progression was objectively measured has not been mentioned in the discussion. Did the authors use only clinical evaluation or was any form of imaging used? The patients detailed in Table 3 represent the minority with aggressive disease, some of whom still die as a result of desmoid despite all the treatments described.

We wholeheartedly support the authors’ statement that ‘clustering of desmoid patients in some specialised referral centres will benefit treatment and follow-up and enables further research into this controversial topic’. Only such centralisation and co-operation between centres will allow any progress in the understanding of these rare tumours and development of effective treatment strategies.
